# Clinical outcomes and perioperative morbidity and mortality following segmental resections of the colon for Crohn’s colitis

**DOI:** 10.1007/s00384-024-04596-w

**Published:** 2024-03-08

**Authors:** Alina-Sophie Kappenberger, Josefine Schardey, Ulrich Wirth, Florian Kühn, Jens Werner, Petra Zimmermann

**Affiliations:** Department of General, Visceral, and Transplant Surgery, Ludwig-Maximilians-University Munich, Marchionini Str. 15, 81377 Munich, Germany

**Keywords:** Crohn’s colitis, Ileocolic resection, Colonic resection, NOD2, Inflammatory bowel disease, Risk factors for recurrence

## Abstract

**Introduction:**

Crohn’s disease (CD) is a chronic inflammatory bowel disease of a multifactorial pathogenesis. Recently numerous genetic variants linked to an aggressive phenotype were identified, leading to a progress in therapeutic options, resulting in a decreased necessity for surgery. Nevertheless, surgery is often inevitable. The aim of the study was to evaluate possible risk factors for postoperative complications and disease recurrence specifically after colonic resections for CD.

**Patients and methods:**

A total of 241 patients who underwent colonic and ileocaecal resections for CD at our instiution between 2008 and 2018 were included. All data was extracted from clinical charts.

**Results:**

Major complications occurred in 23.8% of all patients. Patients after colonic resections showed a significantly higher rate of major postoperative complications compared to patients after ICR (*p* =  < 0.0001). The most common complications after colonic resections were postoperative bleeding (22.2%), the need for revision surgery (27.4%) and ICU (17.2%) or hospital readmission (15%). As risk factors for the latter, we identified time interval between admission and surgery (*p* = 0.015) and the duration of the surgery (*p* = 0.001). Isolated distal resections had a higher risk for revision surgery and a secondary stoma (*p* = 0.019). Within the total study population, previous bowel resections (*p* = 0.037) were identified as independent risk factors for major perioperative complications.

**Conclusion:**

The results indicate that both a complex surgical site and a complex surgical procedure lead to a higher perioperative morbidity in colonic resections for Crohn’s colitis.

## Introduction

Crohn’s disease (CD), first described by Crohn et al. [1] in 1932, is a chronic inflammatory bowel disease leading to discontinuous inflammation of the gastrointestinal (GI) tract [2, 3]. Typically, patients experience symptom-free intervals, alternating with periods of disease activity. In general, disease manifestation can be found throughout the whole GI tract, with the terminal ileum being the most common site. About 25% of patients show an isolated affection of the colon leading to Crohn’s colitis, which needs to be thoroughly differentiated from ulcerative colitis [4].

Chronic recurrent inflammation can lead to disease-specific complications. Stenosis, fistulas, abscesses or inflammatory tumours are common complications that up to 50% of patients might develop over time.

Despite a conservative therapeutical approach with monoclonal antibodies (MAB), disease-modifying antirheumatic drugs (DMARDs) or steroids, being the first choice for patients diagnosed with CD, a large proportion of patients will still need surgical intervention at one point [2–7]. However, surgical procedures are a challenge due to the underlying inflammation and morbidity rates as well as rates for disease recurrence are high [2, 4, 8]. Patient populations have been analysed for possible risk factors leading to higher morbidity in case of CD-associated surgeries. The current literature states duration of the disease, length of the resected intestinal segment, preoperative medication or specific phenotypical findings as possible risk factors for higher perioperative morbidity and mortality [2, 8–10]. The role of underlying genetic alterations such as mutations in the NOD2 gene, which are associated with a more aggressive phenotype of CD, remains controversial [3]. Some authors describe no influence on the postoperative course after ileocolonic resections [2], whereas others show a negative predictive role of the mutation on the perioperative outcome after ileocolectomy [11].

Within the group of CD patients needing surgery, colonic resections are not very common which leads to a lack of information on morbidity and mortality after CD-associated colonic resections.

Therefore, the aim of the current study was to analyse rates for perioperative morbidity and mortality and to evaluate possible risk factors for postoperative complications and disease recurrence specifically in case of colonic resections for CD compared to ileocaecal resections (ICR).

## Material, methods and patients

### Study design

The study was designed as a monocentric, observational cohort study at a single academic reference centre. The study was approved by the Institutional Review Board of the LMU University of Munich (protocol number EK-LMU 18–677). This retrospective cohort study was conducted according to the Strengthening the Reporting of Observational Studies in Epidemiology (STROBE) guidelines.

### Study population

A total of 241 patients with histologically confirmed CD who underwent colonic resection, ileocaecal resection (ICR) or re-resection after ICR including the anastomosis at the Department of General, Visceral and Transplant Surgery at Ludwig-Maximilians-University between 2008 and 2018 were included. Forty-four of the 241 were also included in a previous study of our own group, evaluating the influence of *NOD2* mutations on morbidity and mortality after ICR between 2003 and 2013 [2].

### Data sources

Demographic data, information on medical history, clinical examinations, genetic alterations, pre- and postoperative medical therapy, duration and type of surgery and information on postoperative complications were extracted from the clinical documentation system, clinical charts, endoscopic reports and anaesthesiology reports.

Postoperative complications were defined according to the Clavien-Dindo classification [12]. Minor complications were defined as Clavien-Dindo grade < 3 and major complications as Clavien-Dindo ≥ 3a.

### Outcomes

Overall perioperative morbidity and mortality were defined as primary endpoints. Correlation between morbidity and previous medical therapy, duration of disease, previous surgeries, timing of surgery, diagnosis leading to surgery, type of surgery and underlying genetic mutation as well as the rate of disease recurrence were defined as secondary endpoints.

### Statistical analysis

This study was carried out as an explorative study. For statistical analysis, the SPSS statistical software package (version 27.0, IBM, Chicago, IL) was used.

Descriptive statistical analysis was performed and relations between postoperative morbidity and mortality and the conditions mentioned above were reviewed. The chi-square test or Mann–Whitney *U* test was used for comparative analysis. Significant variables were further entered into multivariate analysis, using logistic regression calculations.

*p* < 0.005 was regarded as statistically significant.

## Results

A total of 241 patients, 127 males (52.5%) and 114 females (47.5%), were included in the study. Mean age at the time of surgery was 42.74 years (± 15.25 years) and mean age at diagnosis of CD was 29.90 years (± 14.47 years), leading to a mean duration of disease of 11.86 years (± 8.63 years) between first diagnosis and current surgery. Mean follow-up after surgery was 68.9 months (± 47.48 months), with data being available for 191 patients (79.3%). Data on previous medical conditions, prior abdominal surgeries and pre- and postoperative medical therapy is available in Table 1.

**Table 1:** Demographic data, clinical characteristics and information on medical therapy of the study population

	*n* (%)
Age at date of surgery* (years)	41.74 (± 15.25)
Age at diagnosis* (years)	29.90 (± 14.46)
Gender	127 (52.5) males 114 (47.5) females
Preexisting medical conditions	114 (47.3)
Previous abdominal surgery	
Overall	148 (61.4)
Bowel resection	92 (38.2)
Appendectomy	44 (18.3)
Preoperative drug therapy	
Overall	186 (77.2)
MAB	108 (44.8)
DMARDs	105 (43.6)
Mesalazine	25 (10.4)
Steroids	86 (35.7)
Postoperative drug therapy	
Overall	156 (64.7)
MAB	112 (46.5)
DMARDs	56 (23.2)
Mesalazine	12 (5.0)
Steroids	47 (19.5)
*NOD2* mutations	19 (7.8)

^*^Median and (standard deviation)

Information on genetic alterations was available for 36 patients and NOD2 mutation was detected in 19 patients, which equals a *NOD2* mutation rate of 7.8% among the whole study population.

89.6% (*n* = 215) of patients underwent elective surgeries, whereas 10.4% (*n* = 25) had an emergency procedure. Data on the primary surgical approach was available for 233 patients. 69.1% (*n* = 161) of procedures were performed primarily with an open approach, and 30.9% (*n* = 72) were performed laparoscopically. The rate of laparoscopic procedures was significantly higher in the case of ICR (*p* < 0.001). Conversion to an open approach was necessary in 22.54% (*n* = 16), 11 times for ICR, in 4 cases of colonic resections and one time for a combined resection (*p* = 0.058). The majority of patients received a handsewn anastomosis (87%, *n* = 167). Twenty-five (13%) anastomoses were stapled, 12 for ICR and 13 for colonic resections (*p* = 0.874).

Mean duration of surgery was 168.2 min (± 86.05 min). Mean time between hospital admission and surgery was 3.59 days (± 4.98 days).

Most frequent indications for surgery were stenosis in 59.9%, septic complications and fistulas in 12.1%, perforation in 7.8% and abscess formation in 7.3%. Less frequent reasons for surgery were conglomerate tumours in 3.4% and carcinomas in 3.9%.

Fifty-six percent (*n* = 135) of all patients included underwent colonic resections, whereas 44% (*n* = 106) underwent ileocaecal resection. Forty-one patients (17%) received a primary stoma, 26 patients due to severe inflammation. Two patients received a stoma as they underwent low anterior resection, and three patients received a definite ostomy due to rectal extirpation. In 10 cases, the reason for a temporary stoma was not documented.

Details on all surgical procedures are shown in Table 2.

**Table 2:** Overview on performed procedures, surgical approaches and anastomotic techniques within the study population

	*n* (%)	*p* value
Ileocaecal resection	106 (44.0)	
Right-sided hemicolectomy	75 (31.1)	
Extended right-sided hemicolectomy	10 (4.2)	
Left-sided hemicolectomy	6 (2.5)	
Extended left-sided hemicolectomy	4 (1.7)	
Sigma resection	6 (2.5)	
Anterior sigma rectal resection	3 (1.2)	
Anterior rectal resection	2 (0.8)	
Low anterior rectal resection	4 (1.7)	
Rectal extirpation	3 (1.2)	
Proctocolectomy	4 (1.7)	
Segmental colonic resections	8 (3.3)	
Combined resections	10 (4.2)	
Laparoscopic procedures		< 0.001*
ICR	56 (53.8)	
Colonic resections	16 (12.4)	
Conversion to open procedure		0.058
ICR	11 (19.6)	
Colonic resections	5 (31.25)	
Stapled anastomosis		0.874
ICR	12 (12.6)	
Colonic resections	13 (13.4)	

*Statistically significant

### Perioperative morbidity and mortality

Out of 241 patients, 75.9% (*n* = 183) had no or only minor complications and 23.1% (*n* = 56) experienced major complications. Overall mortality was 1.25% (*n* = 3) among the whole population.

Patients after colonic resections experienced major complications (34.6%, *n* = 47) significantly more often than patients after ICR (9.4%, *n* = 10), *p* < 0.001. Most common complications were postoperative bleeding, the need for revision surgery and readmission to ICU and/or hospital. Likewise, the latter occurred more frequently after colonic resections, *p* < 0.001. However, there was no difference in anastomotic leaks, abdominal fluid collections, wound infections or necessity for vacuum therapy between the groups. Details are shown in Table 3.

**Table 3:** Distribution of postoperative complications

	Colonic resection, *n* (%)	ICR, *n* (%)	*p* value
Postoperative bleeding	30 (22.2)	2 (1.9)	< 0.001*
Anastomotic bleeding	2	2	
Intrabdominal bleeding	2	0	
Source not documented	26	0	
Woundinfection	28 (20.9)	13 (12.5)	0.890
Vacuum therapy	18 (13.4)	3 (2.9)	0.040
Intraabdominal fluid collection	22 (16.4)	7 (6.7)	0.023
Anastomotic leak	7 (5.2)	6 (5.7)	0.857
Subileus	4 (3.0)	6 (5.8)	0.283
Ileus	4 (3.0)	0 (0.0)	0.073
Re-do procedure	37 (27.4)	7 (6.8)	< 0.001*
Readmission to ICU	23 (17.2)	2 (1.9)	< 0.001*
Readmission to hospital	20 (15.0)	4 (3.9)	< 0.001*
Overal rate of major complications	47 (34.6)	10 (9.4)	< 0.001*

*Statistically significant

With regard to the higher rate of major complications after colonic resections, an univariate analysis revealed the interval between hospital admission and surgery, as well as the duration of the surgical procedure as risk factors for a complicated postoperative course. However, neither pre- nor postoperative medical therapy nor gender, age and preexisting medical conditions showed a significant influence on postoperative morbidity after colonic resections. Indication for surgery, the surgical approach and the urgency of surgery did not show any influence on perioperative morbidity in our analysis (Table 4).

**Table 4:** Univariate analysis for major postoperative complications in colonic resections

	All patients, *n* (%)	No/minor complications, *n* (%)	Major complications, *n* (%)	*p* value
Age at date of surgery* [years]	46.3 [± 13.9]	45.9 [± 12.8]	47.1 [± 15.9]	0.392
Age at diagnosis* (years)	30.0 [± 13.9]	30.7 [± 13.6]	28.7 [± 14.5]	0.890
Gender: female	66 (48.9)	41 (62.1)	25 (37.9)	0.456
Gender: male	69 (51.1)	47 (68.1)	22 (31.9)	
Preexisting medical conditions	74 (54.8)	44 (59.5)	30 (40.6)	0.141
NOD2 mutation	10 (7.4)	6 (60)	4 (40)	0.153
Previous bowel resection	84 (62.2)	55 (65.5)	29 (34.5)	0.976
Preoperative drug therapy	105 (77.8)	69 (65.7)	36 (34.3)	0.710
MAB	63 (46.7)	45 (71.4)	18 (28.6)	0.230
DMARD	47 (34.8)	33 (70.2)	14 (29.8)	0.544
Mesalazine	15 (11.1)	8 (53.3)	7 (46.7)	0.275
Steroids	53 (39.3)	32 (60.4)	21 (39.6)	0.221
Surgical indication				
Stenosis	73 (54.1)	52 (71.3)	21 (28.7)	0.074
Fistula	21 (15.6)	12 (57.1)	9 (42.9)	0.434
Abscess	7 (5.2)	4 (57.1)	3 (42.9)	0.671
Perforation	8 (5.9)	5 (62.5)	3 (37.5)	0.897
Carcinoma	8 (5.9)	4 (50.0)	4 (50.0)	0.372
Conglomerate tumour	7 (5.2)	3 (42.9)	4 (57.1)	0.216
Emergency surgery	12 (89.0)	6 (50.0)	6 (50.0)	0.256
Laparoscopic procedure	16 (11.9)	13 (81.3)	3 (18.7)	0.116
Conversion to open procedure	5 (31.3)	4 (80.0)	1 (20.0)	0.428
Interval between admission and surgery* [days]	4.2 [± 5.9]	2.9 [± 3.3]	6.6 [± 8.4]	0.015**
Duration of surgical procedure* [minutes]	197.1 [± 87.6]	169.0 [± 66.1]	246.7 [± 100.9]	0.001**

^*^Median and (standard deviation)

**Statistically significant

Distal resections (*p* = 0.019) and history of prior bowel resections (*p* = 0.007) emerged as significant risk factors for need of a stoma (univariate analysis). At the same time, distal resections (*p* = 0.0019), interval between hospital admission and surgery (*p* = 0.001) and previous bowel resections (*p* < 0.0001) increased the likelihood for postoperative ICU admission. Distal resections were also detected as a risk factor for postoperative bleeding (*p* < 0.0001).

Within the whole study population, including ICR and colonic resections, the interval between hospital admission (*p* = 0.018; OR 1.23), duration of the surgical procedure ([minutes] *p* = 0.008; OR 1.01) and previous bowel resections (*p* = 0.037; OR 7.75) were identified as independent risk factors for major perioperative complications.

The anastomotic technique used had no influence on perioperative morbidity neither for the whole population (*p* = 0.797), nor for colonic resections alone (*p* = 0.716).

*NOD2* mutation, detected in 7.8% of patients, did not increase perioperative morbidity, with a rate of major complications of 21.1% among patients with *NOD2* mutation and of 32.1% among patients without *NOD2* mutation. However, genetic alterations were only tested in a subgroup of patients.

### Long-term follow-up and disease recurrence

Data on long-term follow-up were available in 191 of 241 patients (79.3%). The mean duration of follow-up was 68.9 months (± 47.48 months). The data revealed a decrease in the rate of long-term postoperative drug therapy from 77.2 to 64.7% (Table 1).

Among the study population, the rate of disease recurrence was 16.3%. There was no difference between ICR (12.5%) and colonic resections (19.1%), *p* = 0.225. The univariate analysis found the duration of ICU stay (*p* = 0.036) to be a risk factor for disease recurrence, whereas the multivariate analysis did not confirm this finding; details are shown in Table 5.

**Table 5:** Univariate and multivariate analysis for disease recurrence in the study population

	All patients, *n* (%)	No disease recurrence, *n* (%)	Disease recurrence, *n* (%)	Univariate analysis, *p* value	Multivariate analysis, *p* value
Age at surgery* [years]	41.7 [± 15.3]	41.4 [± 14.4]	43.1 [± 15.7]	0.598	
Age at diagnosis* [years]	29.7 [± 14.5]	28.9 [± 13.3]	30.7 [± 15.4]	0.542	
Preexisting medical conditions	114 (47.3)	78 (32.4)	12 (4.9)	0.277	
NOD2 mutation	19 (7.9)	16 (6.6)	1 (0.4)	0.545	
Previous bowel resection	92 (38.2)	59 (24.5)	14 (5.8)	0.363	
Interval between admission and surgery* [days]	3.6 [± 4.9]	3.4 [± 4.5]	2.7 [± 2.9]	0.791	
Duration of surgical procedure* [minutes]	168.2 [± 86.1]	166.7 [± 86.7]	178.9 [± 52.3]	0.234	
Duration postoperative ICU stay* [days]	1.7 [± 7.9]	1.1 [± 6.7]	3.3 [± 10.6]	0.036**	0.425
Major postoperative complications	57 (23.7)	12 (4.9)	35 (14.5)	0.049	0.107
Preoperative drug therapy	151 (62.6)	127 (52.7)	24 (9.9)	0.775	
Postoperative drug therapy	133 (55.2)	110 (41.5)	23 (9.5)	0.947	

^*^Median and (standard deviation)

**Statistically significant

## Discussion

Crohn’s disease as one of the two chronic inflammatory bowel diseases most commonly affects the ileocecal region [19]. About one quarter of patients show an isolated involvement of the colon [4]. Based on the frequency of performed surgeries, our study population including 43.98% ileocecal resections, 35.27% right hemicolectomies and 11.57% distal colon resections is representative for this condition and aligns with previous studies [2, 13–15].

In Crohn’s disease, periods of inflammation usually alternate with less severe or no disease activity. Disease activity frequently results in complications such as stenosis, abscess or fistula. The latter inevitably resulted in the need for surgery, despite advancements in medical treatment options. Besides, the short- and long-term results of the LIR!C trial suggest potential benefits for selected Crohn’s patients with early surgical intervention prior to possible complications [17, 18], Therefore, there is an increased emphasis on the outcomes following surgery.

ICR is certainly one of the main surgical procedures among CD patients, and results on possible influencing factors on surgical results have been well described in the literature [13–16]. However, colon resections might be necessary in case of colonic involvement.

Among the whole study population, 75.9% (*n* = 183) experienced no or only minor complications, whereas 23.1% (*n* = 56) had major complications after surgery.

In our study, we found more major complications after colon resections compared to ICR. Especially postoperative bleeding, the need for re-do surgery and readmission to ICU and/or hospital occurred more frequently. However, anastomotic leakage was not increased among colonic resections compared to ICR. Further analysis revealed distal resections as a risk factor for higher perioperative morbidity as well as for re-do procedures, secondary stoma and ICU admission. Presumably due to the small number of cases in our study population, these influences could not be confirmed in the multivariate analysis. Previously, Landerholm and co-workers showed in their study that colonic anastomosis are a significant risk factor for septic complications [26]. These findings suggest that colonic involvement in CD, especially distal involvement, represents a more complicated phenotype, leading, in combination with more complex surgical procedures, to higher rates of complications itself.

Further, we could identify two independent risk factors for high morbidity following colonic resections, the interval between hospital admission and surgery and the duration of surgery. These findings are in accordance with the results by Kanazawa et al. [29]. Likewise, did some groups state a reduced nutritional status, penetrating disease such as abscess or fistula and preoperative steroid use as risk factors for a complicated postoperative course [26–29]. The two risk factors identified in our study, interval to surgery and duration of surgery, support these results as they implicate a possible reduced patient status at hospital admission as well as complex surgical site as possible reason for the delay and a prolonged operating time. Due to evolving conservative therapeutic options, patients with Crohn’s disease are often treated for long periods of time, leading to an immense impairment of their general state of health. Although delay of surgery is used to improve general condition, previous years of chronic inflammation and malabsorption cannot be entirely eliminated. In case of a severe phenotype of CD, possibly involving multiple intestinal segments, especially sufficient nutrition should be focused on, not only during a short time period prior to surgery but also during medical therapy. The latter could possibly lead to a reduction of cases needing surgery in a severe nutritional and inflammatory status.

Additionally, previous bowel resections, which we could identify as a risk factor for major complications after colonic and ileocaecal resections, also often lead to a more complex surgical site. These results are in concordance to the findings by Crowell and colleagues as well as by Duan et al. [27, 29]. Further, a former study from our own group also accounted these findings among patients with isolated ICR [2]. On the other hand, Yamamoto et al. could not confirm a relation between prior intestinal resections and postoperative morbidity [28]. Moreover, a study by Aaltonen and coworkers found emergency procedures to be a risk factor for perioperative complications, which our findings did not confirm [19]. However, our own current study and several previous studies from other groups suggest that the above-described factors, delay of surgery, duration of surgery and emergency procedures, are a surrogate for an underlying severe phenotype of CD. The latter might lead to an increase in perioperative morbidity, especially in combination with complex surgical procedures involving the distal colon [25, 27, 29].

Preoperative medication could not be confirmed as a risk factor for higher perioperative morbidity among patients after colonic resections in our study. Although univariate analysis showed an impact, this effect could not be affirmed in the multivariate analysis. These findings are in line with results by Iesalnieks et al. [21]. However, in the literature, steroid use in general is considered a risk factor for a complicated perioperative course [22, 24]. Bernstein et al. described pre- and postoperative steroid use as a clear risk factor for perioperative complications, especially that long-term steroid therapy is considered to be a relevant factor [20, 22]. As data on duration of steroid therapy was limited in our patient population, we could not perform further analysis regarding this aspect. Regarding the perioperative use of immunomodulatory drugs, there is still a controversial discussion present [24]. Our current study, like results by Kumar and coworkers, did not show any influence on perioperative morbidity [23]. Yet, other groups, like Landerholm et al., showed a significant impact of thiopurine use on the postoperative rate of anastomotic insufficiencies. Fumery et al., as well as Shah et al., also showed no association between preoperative use of monoclonal antibodies and postoperative complications [31, 32]. In general, results regarding the influence of immunomodulatory drugs on the perioperative outcome are heterogeneous.

Information on genetic alterations was only available for a subgroup of patients, and the rate for *NOD2* mutations was 7.8% in the whole population. Therefore, possible conclusions are limited. Out of all patients with a detected *NOD2* mutation, only 4 (1.7%) developed major complications, whereas the rest did not have any or only minor problems postoperatively. This proportion is below that of the overall study population, even if the significance of such small numbers of patients is limited. Comparable results were shown in a previous study from our own group, where *NOD2* did not influence the perioperative course after ICR [2], However, there are studies published in the literature that showed an influence of the underlying germline mutation on perioperative outcome [11, 33, 34].

Concerning disease recurrence after resections, our results suggest that a prolonged ICU stay is a possible risk factor. Major complications alone did not lead to an increased risk for disease recurrence; however, numbers for this subgroup were small. Concerning this point, our results differ to some extent from previous studies, which identified surgical complications as risk factors for disease recurrence [22, 30]. Yet, a prolonged ICU stay due to different reasons suggests that a postoperative delay of a disease-specific medical therapy leads to higher recurrence rates.

Although the results presented identified distinct risk factors for more complications after colonic resections for CD, there are some limitations of this study. The retrospective design and therefore heterogeneity of follow-up data as well as data only from a single centre limit the generalisability of our results. However, the size of the study population allowed valid subgroup analysis leading to a more detailed understanding of perioperative courses in case of colonic resections for Crohn’s colitis.

## Conclusion

Although results of the presented study are to some extent limited by the size of subgroups, we believe that our results clearly show that colonic resections for CD are a challenging situation in surgery. Higher rates for morbidity after colonic resections than after ICR and higher rates for complications after distal resections indicate that a severe phenotype in combination with a complex surgical procedure is a relevant risk factor for a complicated perioperative course. Prolonged ICU stays were associated with higher rates of recurrence, indicating that a possible delay of prophylactic medical treatment adds to this trend.

## Data Availability

Raw data for this study are not publicly available to preserve patients’ privacy under the European Data Protection Regulation.

## References

[1] Crohn BB, Ginzburg L, Oppenheimer GD (1984) Landmark article Oct 15, 1932. Regional ileitis. A pathological and clinical entity. JAMA 251(1):73–79. 10.1001/jama.1984.03340250053024 6361290 10.1001/jama.251.1.73

[2] Schardey J, Zehl S, Kappenberger AS, Zimmermann P, Beigel F, Schiergens TS, Kasparek MS, Kühn F, Werner J, Wirth U (2022) It is not NOD2 - genetic and clinical risk factors for postoperative complications following ileocolic resection in Crohn’s disease. Int J Colorectal Dis 37(8):1901–1908. 10.1007/s00384-022-04223-6. (Epub 2022 Aug 1. PMID: 35913516; PMCID: PMC9388399) 35913516 10.1007/s00384-022-04223-6 PMC9388399

[3] Torres J, Mehandru S, Colombel JF, Peyrin-Biroulet L (2017) Crohn’s disease. Lancet 389(10080):1741–1755. 10.1016/S0140-6736(16)31711-1. (Epub 2016 Dec 1 PMID: 2791465) 27914655 10.1016/S0140-6736(16)31711-1

[4] Gajendran M, Loganathan P, Catinella AP, Hashash JG (2018) A comprehensive review and update on Crohn’s disease. Dis Mon 64(2):20–57. 10.1016/j.disamonth.2017.07.001. (Epub 2017 Aug 18 PMID: 28826742) 28826742 10.1016/j.disamonth.2017.07.001

[5] Peyrin-Biroulet L, Loftus EV Jr, Colombel JF, Sandborn WJ (2010) The natural history of adult Crohn’s disease in population-based cohorts. Am J Gastroenterol 105(2):289–297. 10.1038/ajg.2009.579. (Epub 2009 Oct 27 PMID: 19861953) 19861953 10.1038/ajg.2009.579

[6] Toh JW, Stewart P, Rickard MJ, Leong R, Wang N N, Young CJ (2016) Indications and surgical options for small bowel, large bowel and perianal Crohn’s disease. World J Gastroenterol 22(40):8892–8904. 10.3748/wjg.v22.i40.8892. (PMID: 27833380; PMCID: PMC5083794) 27833380 10.3748/wjg.v22.i40.8892 PMC5083794

[7] Frolkis AD, Dykeman J, Negrón ME, Debruyn J, Jette N, Fiest KM, Frolkis T, Barkema HW, Rioux KP, Panaccione R, Ghosh S, Wiebe S, Kaplan GG (2013) Risk of surgery for inflammatory bowel diseases has decreased over time: a systematic review and meta-analysis of population-based studies. Gastroenterology 145(5):996–1006. 10.1053/j.gastro.2013.07.041. (Epub 2013 Jul 27 PMID: 23896172) 23896172 10.1053/j.gastro.2013.07.041

[8] Hossne RS, Sassaki LY, Baima JP, Meira Júnior JD, Campos LM (2018) Analysis of risk factors and postoperative complications in patients with Crohn’s disease. Arq Gastroenterol 55(3):252–257. 10.1590/S0004-2803.201800000-63. (PMID: 30540087) 30540087 10.1590/S0004-2803.201800000-63

[9] Abou Khalil M, Abou-Khalil J, Motter J, Vasilevsky CA, Morin N, Ghitulescu G, Boutros M (2019) Immunosuppressed patients with Crohn’s disease are at increased risk of postoperative complications: results from the ACS-NSQIP database. J Gast-rointest Surg 23(6):1188–1197. 10.1007/s11605-019-04186-0. (Epub 2019 Mar 18 PMID: 30887300) 10.1007/s11605-019-04186-0 30887300

[10] Hefaiedh R, Sabbeh M, Miloudi N, Ennaifer R, Romdhane H, Belhadj N, Gharbi L, Khalfallah T (2015) Surgical treatment of Crohn’s disease: indications, results and predictive factors of recurrence and morbidity. Tunis Med 93(6):356–360 (PMID: 26644097) 26644097

[11] Kline BP, Weaver T, Brinton DL Jr, Deiling S, Yochum GS, Berg AS, Koltun WA (2020) Clinical and genetic factors associated with complications after Crohn’s ileocolectomy. Dis Colon Rectum 63(3):357–364. 10.1097/DCR.0000000000001574. (PMID: 32045400) 32045400 10.1097/DCR.0000000000001574

[12] Dindo D, Demartines N, Clavien PA (2004) Classification of surgical complications: a new proposal with evaluation in a cohort of 6336 patients and results of a survey. Ann Surg 240(2):205–213. 10.1097/01.sla.0000133083.54934.ae.PMID:15273542;PMCID:PMC1360123 15273542 10.1097/01.sla.0000133083.54934.ae PMC1360123

[13] Celentano V, Pellino G, Spinelli A, Selvaggi F; SICCR Current status of Crohn’s disease surgery collaborative; Celentano V, Pellino G, Rottoli M, Poggioli G, Sica G, Giglio MC, Campanelli M, Coco C, Rizzo G, Sionne F, Colombo F, Sampietro G, Lamperti G, Foschi D, Ficari F, Vacca L, Cricchio M, Giudici F, Selvaggi L, Sciaudone G, Peltrini R, Manfreda A, Bucci L, Galleano R, Ghazouani O, Zorcolo L, Deidda S, Restivo A, Braini A, Di Candido F, Sacchi M, Carvello M, Martorana S, Bordignon G, Angriman I, Variola A, Di Ruscio M, Barugola G, Geccherle A, Tropeano FP, Luglio G, Tanzanu M, Sasia D, Migliore M, Giuffrida MC, Marrano E, Moretto G, Impellizzeri H, Gallo G, Vescio G, Sammarco G, Terrosu G, Calini G, Bondurri A, Maffioli A, Zaffaroni G, Resegotti A, Mistrangelo M, Allaix ME, Botti F, Prati M, Boni L, Perotti S, Mineccia M, Giuliani A, Romano L, Graziano GMP, Pugliese L, Pietrabissa A, Delaini G, Spinelli A, Selvaggi F, On behalf of the Italian Society of Colorectal Surgery SICCR (2021) Anastomosis configuration and technique following ileocaecal resection for Crohn’s disease: a multicentre study. Updates Surg 73(1):149–156. 10.1007/s13304-020-00918-z. (Epub 2021 Jan 6. PMID: 33409848) 33409848 10.1007/s13304-020-00918-z

[14] Garofalo E, Lucarini A, Flashman KG, Celentano V (2019) A positive proximal resection margin is associated with anastomotic complications following primary ileocaecal resection for Crohn’s disease. Int J Colorectal Dis 34(9):1585–1590. 10.1007/s00384-019-03358-3. (Epub 2019 Aug 3 PMID: 31377853) 31377853 10.1007/s00384-019-03358-3

[15] Reynolds IS, Doogan KL, Ryan ÉJ, Hechtl D, Lecot FP, Arya S, Martin ST (2021) Surgical strategies to reduce postoperative recurrence of Crohn’s disease after ileocolic resection. Front Surg 17(8). 10.3389/fsurg.2021.804137. (PMID: 34977147; PMCID: PMC8718441) 10.3389/fsurg.2021.804137 PMC8718441 34977147

[16] 2015 European Society of Coloproctology collaborating group (2017) Risk factors for unfavourable postoperative outcome in patients with Crohn’s disease undergoing right hemicolectomy or ileocaecal resection An international audit by ESCP and S-ECCO. Colorectal Dis. 10.1111/codi.13889 10.1111/codi.13889 28913968

[17] Ponsioen CY, de Groof EJ, Eshuis EJ, Gardenbroek TJ, Bossuyt PMM, Hart A, Warusavitarne J, Buskens CJ, van Bodegraven AA, Brink MA, Consten ECJ, van Wagensveld BA, Rijk MCM, Crolla RMPH, Noomen CG, Houdijk APJ, Mallant RC, Boom M, Marsman WA, Stockmann HB, Mol B, de Groof AJ, Stokkers PC, D’Haens GR, Bemelman WA, LIR!C study group (2017) Laparoscopic ileocaecal resection versus infliximab for terminal ileitis in Crohn’s disease: a randomised controlled, open-label, multicentre trial. Lancet Gastroenterol Hepatol 2(11):785–792. 10.1016/S2468-1253(17)30248-0. (Epub 2017 Aug 31. Erratum. In: Lancet Gastroenterol Hepatol) 28838644 10.1016/S2468-1253(17)30248-0

[18] Stevens TW, Haasnoot ML, D’Haens GR, Buskens CJ, de Groof EJ, Eshuis EJ, Gardenbroek TJ, Mol B, Stokkers PCF, Bemelman WA, Ponsioen CY, LIR!C study group, (2020) Laparoscopic ileocaecal resection versus infliximab for terminal ileitis in Crohn’s disease: retrospective long-term follow-up of the LIR!C trial. Lancet Gastroenterol Hepatol 5(10):900–907. 10.1016/S2468-1253(20)30117-5. (Epub 2020 Jun 30. PMID: 32619413) 32619413 10.1016/S2468-1253(20)30117-5

[19] Aaltonen G, Keränen I, Carpelan-Holmström M, Lepistö A (2018) Risk factors for anastomotic recurrence after primary ileocaecal resection in Crohn’s disease. Eur J Gastroenterol Hepatol 30(10):1143–1147. 10.1097/MEG.0000000000001206. (PMID: 30024490) 30024490 10.1097/MEG.0000000000001206

[20] Bernstein CN, Regueiro M (2023) Postoperative Crohn’s disease. J Clin Gastroenterol 57(8):749–753. 10.1097/MCG.0000000000001865. (PMID: 37224283) 37224283 10.1097/MCG.0000000000001865

[21] Iesalnieks I, Spinelli A, Frasson M, Di Candido F, Scheef B, Horesh N, Iborra M, Schlitt HJ, El-Hussuna A (2018) Risk of postoperative morbidity in patients having bowel resection for colonic Crohn’s disease. Tech Coloproctol 22(12):947–953. 10.1007/s10151-018-1904-0. (Epub 2018 Dec 12 PMID: 30543038) 30543038 10.1007/s10151-018-1904-0

[22] Adamina M, Bonovas S, Raine T, Spinelli A, Warusavitarne J, Armuzzi A, Bachmann O, Bager P, Biancone L, Bokemeyer B, Bossuyt P, Burisch J, Collins P, Doherty G, El-Hussuna A, Ellul P, Fiorino G, Frei-Lanter C, Furfaro F, Gingert C, Gionchetti P, Gisbert JP, Gomollon F, González Lorenzo M, Gordon H, Hlavaty T, Juillerat P, Katsanos K, Kopylov U, Krustins E, Kucharzik T, Lytras T, Maaser C, Magro F, Marshall JK, Myrelid P, Pellino G, Rosa I, Sabino J, Savarino E, Stassen L, Torres J, Uzzan M, Vavricka S, Verstockt B, Zmora O (2020) ECCO Guidelines on therapeutics in Crohn’s disease: surgical treatment. J Crohns Colitis 14(2):155–168. 10.1093/ecco-jcc/jjz187. (PMID: 31742338) 31742338 10.1093/ecco-jcc/jjz187

[23] Kumar A, Auron M, Aneja A, Mohr F, Jain A, Shen B (2011) Inflammatory bowel disease: perioperative pharmacological considerations. Mayo Clin Proc 86(8):748–757. 10.4065/mcp.2011.0074. (PMID: 21803957; PMCID: PMC3146375) 21803957 10.4065/mcp.2011.0074 PMC3146375

[24] Iesalnieks I, Agha A, Dederichs F, Schlitt HJ (2022) Bowel resections for Crohn’s disease: developments over the last three decades. Z Gastroenterol 60(6):927–936. 10.1055/a-1482-9147 34161989 10.1055/a-1482-9147

[25] Landerholm K, Kalman D, Wallon C, Myrelid P (2019) Immunomodulators: friends or enemies in surgery for Crohn’s disease? Curr Drug Targets 20(13):1384–1398. 10.2174/1389450120666190617163919. (PMID: 31237212) 31237212 10.2174/1389450120666190617163919

[26] Alves A, Panis Y, Bouhnik Y, Pocard M, Vicaut E, Valleur P (2007) Risk factors for intra-abdominal septic complications after a first ileocecal resection for Crohn’s disease: a multivariate analysis in 161 consecutive patients. Dis Colon Rectum 50(3):331–336. 10.1007/s10350-006-0782-0. (PMID: 17252288) 17252288 10.1007/s10350-006-0782-0

[27] Crowell KT, Messaris E (2015) Risk factors and implications of anastomotic complications after surgery for Crohn’s disease. World J Gastrointest Surg 7(10):237–242. 10.4240/wjgs.v7.i10.237. (PMID: 26523211; PMCID: PMC4621473) 26523211 10.4240/wjgs.v7.i10.237 PMC4621473

[28] Yamamoto T, Allan RN, Keighley MR (2000) Risk factors for intra-abdominal sepsis after surgery in Crohn’s disease. Dis Colon Rectum 43(8):1141–1145. 10.1007/BF02236563. (PMID: 10950014) 10950014 10.1007/BF02236563

[29] Kanazawa A, Yamana T, Okamoto K, Sahara R (2012) Risk factors for postoperative intra-abdominal septic complications after bowel resection in patients with Crohn’s disease. Dis Colon Rectum 55(9):957–962. 10.1097/DCR.0b013e3182617716. (PMID: 22874602) 22874602 10.1097/DCR.0b013e3182617716

[30] Bachour SP, Shah RS, Rieder F, Qazi T, Achkar JP, Philpott J, Lashner B, Holubar SD, Lightner AL, Barnes EL, Axelrad J, Regueiro M, Click B, Cohen BL (2022) Intra-abdominal septic complications after ileocolic resection increases risk for endoscopic and surgical postoperative Crohn’s disease recurrence. J Crohns Colitis 16(11):1696–1705. 10.1093/ecco-jcc/jjac078. (PMID: 35705188; PMCID: PMC9924045) 35705188 10.1093/ecco-jcc/jjac078 PMC9924045

[31] Shah RS, Bachour S, Jia X, Holubar SD, Hull TL, Achkar JP, Philpott J, Qazi T, Rieder F, Cohen BL, Regueiro MD, Lightner AL, Click BH (2021) Hypoalbuminaemia, not biologic exposure, is associated with postoperative complications in Crohn’s disease patients undergoing ileocolic resection. J Crohns Colitis 15(7):1142–1151. 10.1093/ecco-jcc/jjaa268. (PMID: 33388775; PMCID: PMC8427722) 33388775 10.1093/ecco-jcc/jjaa268 PMC8427722

[32] Guo Z, Cao L, Guo F, Gong J, Li Y, Gu L, Zhu W, Li J (2017) The presence of postoperative infectious complications is associated with the risk of early postoperative clinical recurrence of Crohn’s disease. World J Surg 41(9):2371–2377. 10.1007/s00268-017-4026-3. (PMID: 28508235) 28508235 10.1007/s00268-017-4026-3

[33] Germain A, Guéant RM, Chamaillard M, Allen PB, Bresler L, Guéant JL, Peyrin-Biroulet L (2016) NOD2 gene variant is a risk factor for postoperative complications in patients with Crohn’s disease: a genetic association study. Surgery 160(1):74–80. 10.1016/j.surg.2016.01.013. (Epub 2016 Mar 2 PMID: 26946932) 26946932 10.1016/j.surg.2016.01.013

[34] Giudici F, Cavalli T, Luceri C, Russo E, Zambonin D, Scaringi S, Ficari F, Fazi M, Amedei A, Tonelli F, Malentacchi C (2021) Long-term follow-up, association between CARD15/NOD2 polymorphisms, and clinical disease behavior in Crohn’s disease surgical patients. Mediators Inflamm 24(2021):8854916 10.1155/2021/8854916 PMC7932801 33708009

